# Efficacy of Dolutegravir Plus Lamivudine in People With TB/HIV Co‐Infection Using a Rifampicin or Rifabutin‐Based Regimen: A Retrospective Observational Case Series

**DOI:** 10.1002/iid3.70381

**Published:** 2026-03-22

**Authors:** Jinhong He, Xiangxi He, Xiaoxin Xie, Yanhua Fu, Xingxing Luo, Yinshuang Peng, Bin Xu, Hai Long

**Affiliations:** ^1^ Infection Department Guiyang Public Health Clinical Center Guiyang Guizhou China

**Keywords:** dolutegravir, efficacy, HIV‐1 infection, safety, tuberculosis

## Abstract

**Introduction:**

Co‐infection with tuberculosis (TB) is the leading cause of death in individuals infected with human immunodeficiency virus (HIV)‐1. Dolutegravir and lamivudine (DTG + 3TC) has recently been recommended as the preferred first‐line regimen for the treatment of new and treatment‐experienced HIV‐infected patients. The primary objective of this study was to determine the efficacy and safety of DTG (50 mg) + 3TC (300) mg in HIV‐positive antiretroviral therapy (ART)‐naïve patients with TB who received a rifampicin‐ or rifabutin‐based treatment regimen and characterize viral suppression rates at week 48.

**Methods:**

A single‐center retrospective observational case series, spanning January 1, 2021 to March 1, 2023, was conducted in Guiyang Public Health Treatment Center. Of 46 TB/HIV co‐infected patients received DTG + 3TC or DTG/3TC, a total of 42 patients were finally enrolled in the study. The outcomes of interest were successful TB treatment, viral load (VL) suppression, and immunological and biochemical indexes.

**Results:**

All people with HIV infection (PWH) underwent at least 48 weeks of follow‐up; all TB treatments were successful. A total of seven PWH (100%) achieved viral suppression (VL < 50 copies/mL) from a baseline VL greater than 500,000 copies/mL. Among the PWH who started DTG + 3TC after initiating the rifabutin‐based anti‐TB regimen, 31 (73.8%) achieved viral suppression by week 24. CD4 + T‐cell counts were greatly improved after antiretroviral treatment. The CD4 + /CD8+ ratio increased by 0.38 (*p* < 0.001). Total cholesterol, and high‐density lipoprotein cholesterol (*p* < 0.05). There were no significant changes in body mass index, low‐density lipoprotein cholesterol, and triglyceride levels from baseline to week 48 (*p* > 0.05). No serious adverse events were observed.

**Conclusion:**

This case series preliminarily validated the efficacy of DTG + 3TC when combined with rifabutin‐based anti‐TB regimens in patients with TB and HIV.

## 1 Introduction

Patients with human immunodeficiency virus (HIV) infection or acquired immunodeficiency syndrome (AIDS) can develop opportunistic infections due to an impaired immune system. Tuberculosis (TB)/HIV co‐infection is a common opportunistic infection and important factor influencing disease progression in people with HIV infection (PWH) [1, 2]. It is also the main cause of death in PWH [3]. One report [4] showed that there were an estimated 10.6 million new TB cases globally as of 2021, with an incidence rate of 134/100,000. The prevalence of TB varies widely from country to country, with 30 high‐TB‐burden countries accounting for 87% of all estimated cases globally and 7.1% in China. Mortality among patients with untreated TB is as high as 50%. However, with the newly recommended treatment regimen duration of 4–6 months, 85% of patients can be cured [3, 4].

Dolutegravir (DTG) has recently been recommended as the preferred first‐line regimen for treating new and treatment‐experienced HIV patients [5]. DTG is an important substrate of the cytochrome P450 (CYP) system. Rifamycins are potent inducers of the CYP3A4 isoform, leading to subtherapeutic concentrations of antiretroviral drugs [6], whereas rifabutin is a weak inducer of CYP3A4. Pharmacokinetic studies in healthy adults showed that rifampicin reduced DTG concentrations by > 50% and rifabutin reduced them by 30%. Rifamycins are the cornerstone of first‐line antituberculosis drugs and widely used. The potential drug‐drug interaction between rifamycins and DTG is still a clinical and public health concern. However, TB/HIV co‐infection requires lifelong highly active antiretroviral therapy (ART) and TB drugs treatment; adverse drug reactions can be prominent with long‐term exposure to multiple drugs. Toxic accumulation, drug‐drug interactions, pill burden, and drug resistance have become increasingly prominent, which have adverse effects on patients' compliance, mental health, and quality of life, as well as affecting the efficacy for patients with tuberculosis. To further improve the quality of life of patients and reduce the long‐term impact of ART drugs, many simplified treatment regimens have been developed worldwide, including two‐drug, single‐drug, and intermittent regimens. A variety of simplified two‐drug regimens have been recommended by major guidelines for different patients (including first‐line preferred) [7]. The 2019 European AIDS Clinical Society Conference recommended DTG/3TC as the first‐line treatment in newly diagnosed patients. Large‐scale clinical trials and real‐world data have confirmed that the DTG + 3TC regimen is non‐inferior to the traditional three‐drug regimen, and reduces the drug burden, the risk of adverse events and drug interactions [8]. Although there have been several large‐scale multicenter randomized controlled trials of the DTG + 3TC simplified dual regimen worldwide to confirm its efficacy and safety [7, 8], real‐world data studies are limited, especially in patients with HIV/AIDS complicated with TB. This study evaluated the efficacy and safety of the DTG + 3TC regimen for 24 and 48 weeks in patients with TB/HIV to explore the feasibility and significance of this regimen in the clinical management of TB/HIV.

## 2 Materials and Methods

### 2.1 Study Design and Participants

This study was a single‐center retrospective observational case series conducted at the Guiyang Public Health Clinical Center (Guiyang, China). Patients diagnosed with tuberculosis (TB) and HIV co‐infection who initiated a dolutegravir plus lamivudine (DTG + 3TC) dual antiretroviral regimen between January 1, 2021 and March 1, 2023 were consecutively screened.

Inclusion criteria were: (1) confirmed HIV‐1 infection based on positive screening and confirmatory tests; (2) diagnosis of active TB (pulmonary and/or extrapulmonary) according to international diagnostic criteria; (3) initiation of DTG + 3TC or DTG/3TC as first‐line antiretroviral therapy. Exclusion criteria included: (1) hepatitis B surface antigen positivity; (2) pregnancy; (3) presence of baseline M184V resistance mutation; (4) non‐tuberculous infections.

The study was approved by the Institutional Review Board of Guiyang Public Health Clinical Center (Approval No. 202206). Written informed consent was obtained from all participants prior to enrollment.

### 2.2 Antiretroviral and Anti‐Tuberculosis Treatment

All participants received a dual antiretroviral regimen consisting of dolutegravir (DTG) and lamivudine (3TC), administered either as a fixed‐dose combination tablet or as two separate agents. Anti‐tuberculosis regimens were prescribed in accordance with national TB treatment guidelines and consisted of combinations of isoniazid, rifampicin or rifabutin, ethambutol, and pyrazinamide, with or without fluoroquinolones. When co‐administered with rifampicin, DTG was dosed at 50 mg twice daily. When rifabutin was used, DTG was administered at 50 mg once daily. Adjustments between rifampicin‐based and rifabutin‐based regimens were made based on drug–drug interaction considerations and clinical judgment.

### 2.3 Outcome Measures

Primary outcomes included: (1) virological suppression, defined as plasma HIV‐1 RNA < 50 copies/mL; (2) immunological recovery, assessed by CD4⁺ T‐cell count and CD4⁺/CD8⁺ ratio; (3) safety outcomes, including renal and metabolic parameters; (4) tuberculosis treatment outcomes, evaluated at 6, 9, and 12 months. Assessments were performed at baseline and at 24 and 48 weeks after initiation of DTG + 3TC therapy.

### 2.4 Laboratory Assessments

#### 2.4.1 HIV‐1 RNA Quantification

Plasma HIV‐1 RNA levels were quantified using the COBAS TaqMan HIV‐1 Test on a COBAS TaqMan 48 analyzer (Roche Diagnostics, Switzerland), following the manufacturer's instructions. The lower limit of detection was 20 copies/mL.

#### 2.4.2 Flow Cytometric Analysis of T‐Cell Subsets

Peripheral blood samples were collected in EDTA‐anticoagulated tubes and processed within 24 h. CD4⁺ and CD8⁺ T‐cell subsets were quantified by flow cytometry. Cells were stained with fluorochrome‐conjugated monoclonal antibodies against CD3, CD4, and CD8. Data acquisition was performed on a BD flow cytometer, and analyses were conducted using FlowJo software (BD Biosciences). Lymphocytes were identified based on forward‐ and side‐scatter characteristics, followed by gating of CD3⁺ T cells. CD4⁺ and CD8⁺ T‐cell populations were analyzed within the CD3⁺ gate. Results were expressed as absolute counts (cells/µL) and as the CD4⁺/CD8⁺ ratio.

#### 2.4.3 Biochemical and Renal Parameters

Routine biochemical parameters, including serum creatinine, uric acid, and lipid profiles, were measured using automated clinical chemistry analyzers at the hospital laboratory. Renal function was evaluated using the estimated glomerular filtration rate (eGFR) calculated with the CKD‐EPI equation. Renal dysfunction was defined as an eGFR < 90 mL/min/1.73 m² and/or evidence of persistent renal impairment for at least 3 months.

### 2.5 Tuberculosis Treatment Assessment

Tuberculosis treatment outcomes were evaluated using microbiological, radiological, and clinical assessments. Sputum smear microscopy, mycobacterial culture, and imaging studies were performed at baseline and during follow‐up. Treatment outcomes, including cure and treatment completion, were determined at 6, 9, and 12 months according to national tuberculosis management guidelines.

### 2.6 Statistical Analysis

Statistical analyses were performed using SPSS version 20.0 (IBM Corp., Armonk, NY, USA). Continuous variables were assessed for normality. Normally distributed variables were expressed as mean ± standard deviation and compared using paired or independent‐sample *t*‐tests. Non‐normally distributed variables were summarized as median (interquartile range) and compared using the Wilcoxon signed‐rank test. Categorical variables were presented as counts and percentages. A two‐sided *p* value < 0.05 was considered statistically significant.

## 3 Results

### 3.1 Study Populations and Examination Results

**Figure 1:**
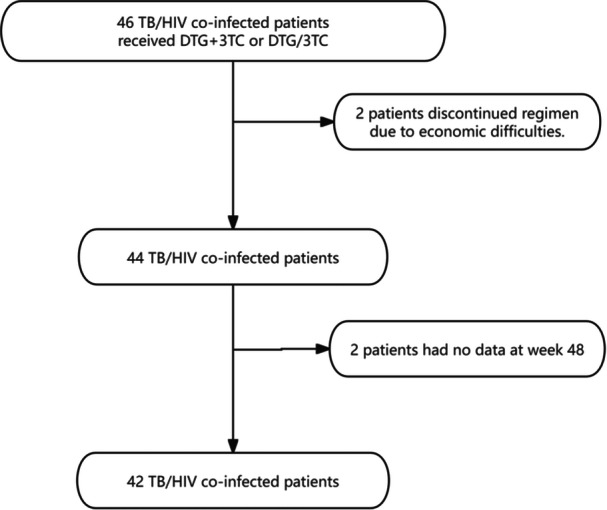
Flowchart of TB/HIV co‐infected patients receiving dolutegravir plus lamivudine.

The patient selection process is shown in Figure 1. Of 46 patients co‐infected with TB/HIV who received DTG + 3TC or DTG/3TC, a total of 42 patients were finally enrolled in the study. The mean age of the patients was 49.31 ± 17.59 years; 80.6% of the patients were male and 19.4% were female. Twelve patients had chronic diseases. CD4 + T‐cell count < 200 cells/mL was found in 85.7% of patients. Baseline HIV RNA levels were < 100,000 cp/mL in 35.7% of patients and ≥ 500,000 cp/mL in 16.7% of patients. Forty patients had HIV drug resistance test results before treatment; there were no M184V drug resistance mutations; and two patients had missing data. The ART regimen was DTG/3TC + DTG (50 mg) in 23.8% of patients and DTG/3TC or DTG + 3TC in 76.2% of patients. A total of 33.3% of the patients had other opportunistic infections detected on chest and abdominal CT and head magnetic resonance imaging (MRI) (Table 1).

**Table 1:** Baseline characteristics of the patient sample (*n* = 42).

Characteristic	ART‐naïve (*N* = 42)
Sex, *n* (%)	
Male	29 (80.6)
Female	13 (19.4)
Age median (mean ± SD), year	49.31 ± 17.59
Age ≥ 60 years, *n* (%)	32 (88.9)
CD4 T‐cell count	
Median (IQR), cells/μL	64.0 (35.50, 139.00)
< 200 cells/µL, *n* (%)	36 (85.7)
≥ 200 cells/µL, *n* (%)	6 (14.3)
CD4 + /CD8 + , median (IQR)	0.19 (0.12, 0.29)
HIV‐1 RNA, copies/mL, *n* (%)	
< 100,000	15 (35.7)
100,000–499,999	20 (47.6)
≥ 500,000	7 (16.7)
CKD‐EPI Scr ≤ 60 mL/min, n (%)	7 (16.7)
BMI (mean ± SD), kg/m^2^	19.60 ± 2.85
WBC (median(IQR)), × 10⁹/L	3.96 (2.86, 6.67)
Hb (mean ± SD), g/L	96.02 ± 27.25
PLT (median (IQR)), × 10⁹/L	141.00 (87.50, 252.00)
LDH (U/L), median (IQR)	223.00 (192.00, 271.00)
CRP, median (IQR), mg/L	44.57 (15.28, 67.77)
ESR, median (IQR), mm/h	71.00 (32.00, 101.50)
IRIS, *n* (%)	11 (26.2) (26.2)
CD4 ≥ 200	0
CD4 < 200	12 (28.57)
Comorbidities, *n* (%)	
Cardiovascular and cerebrovascular diseases	6 (6.0)
Diabetes	2 (4.7)
High blood pressure	2 (4.7)
Renal impairment	2 (4.7)
Other opportunistic infectious diseases	14 (33.3)
IRIS	14 (28.6)
Imageological examination	
The main chest CT findings	
cavity	6 (14.3)
Fibrous strip or caseous necrosis	9 (21.4)
Nodular lesions	9 (21.4)
Millet grain sample	18 (42.9)
Concomitant chest CT images	
enlargement of lymph nodes	8 (19.0)
hydrothorax	19 (45.2)
hydropericardium	11 (26.2)
pleural thickening	14 (33.3)
Head MIR	
Focal cerebral ischemia	11 (26.2)
Normal	30 (74.1)
Not detected	1 (2.4)

Abbreviations: 3TC, lamivudine; AIDS, acquired immunodeficiency syndrome; ART, antiretroviral treatment; CKD‐EPI Scr, A equation of chronic kidney disease epidemiology collaboration base serum creatinine; CRP, C‐reactive protein; DTG, dolutegravir; ESR, erythrocyte sedimentation rate; Hb, hemoglobin; LDH, lactic dehydrogenase; PLT, platelet; WBC, White blood cell.

### 3.2 Virological Suppression

**Figure 2:**
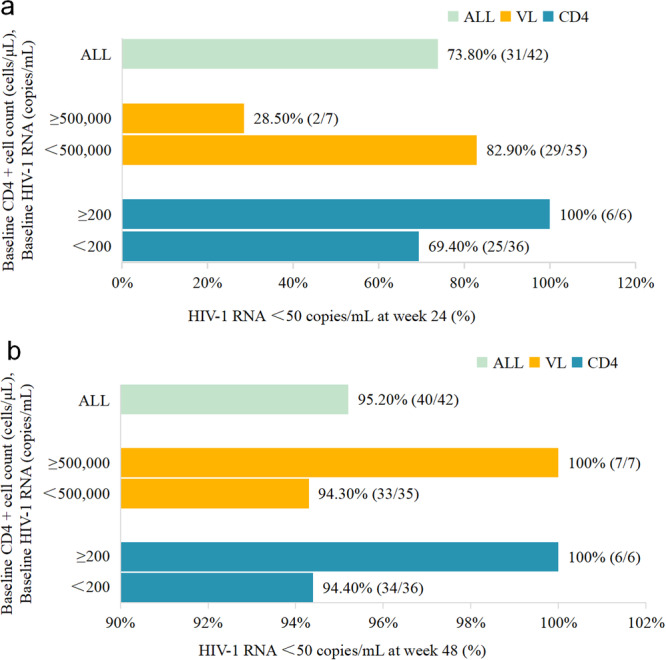
Analysis of patients with an HIV viral load < 50 copies/mL at week 24 and week 48. (a) Evaluation of patients with an HIV viral load < 50 copies/mL at week 24. (b) Evaluation of patients with an HIV viral load < 50 copies/mL at week 48. “ALL” means the analysis of the proportion of patients with VL < 50 copies/mL independent of the CD4+ cell count.

The HIV viral suppression rate at 24 weeks was 73.8%; the viral suppression rates at 24 weeks were 28.5% and 82.9% in the groups with HIV RNA ≥ 500,000 cp/mL and HIV RNA < 500,000 cp/mL, respectively. The viral suppression rates in the groups with CD4 + T‐cell ≥ 200/mL and CD4 + T‐cell < 200/mL at 24 weeks were 100.0% and 69.4%, respectively (Figure 2a).

The 48‐week HIV viral suppression rate was 95.2%; the 48‐week HIV viral suppression rate was 100.0% and 94.3% for the groups with HIV RNA ≥ 500,000 cp/mL and HIV RNA < 500,000 cp/mL, respectively. The viral suppression rates the groups with CD4 + T‐cells ≥ 200/mL and CD4 + T <‐cells 200/mL at 48 weeks were 100.0% and 94.4%, respectively (Figure 2b).

### 3.3 Immunological Level

The CD4 + T‐cell count and CD4 + /CD8 + T‐cell ratio were significantly higher at 24 (Table 2) and 48 (Table 3) weeks than those at baseline (all, *p* < 0.05).

**Table 2:** Changes in biochemical indexes between baseline and Week 24.

Indexes	Baseline	Week 24	*t/z*	*p* value
BMI, kg/m^2^, mean ± SD	19.60 ± 2.85	19.82 ± 2.79	−0.359	0.720
CD4, cells/μL, median (IQR)	64.50 (35.75, 144.00)	166.00 (135.25, 246.50)	−4.420	< 0.001
CD4/CD8, median (IQR)	0.20 (0.12, 0.29)	0.32 (0.20, 0.46)	−3.320	0.001
Scr, μmoL/L, median (IQR)	57.25 (42.00, 65.25)	67.65 (60.00, 92.85)	−3.718	< 0.001
CKD‐EPI Scr, mL/min, median (IQR)	106.55 (71.55, 152.70)	78.30 (53.45, 109.00)	−2.532	0.011
Uric Acid, μmoL/L, median (IQR)	282.50 (204.25, 361.00)	386.50 (322.00, 475.75)	−3.936	< 0.001
TG, mmol/L, median (IQR)	1.78 (1.18, 2.12)	1.84 (1.39, 2.60)	−0.913	0.361
TC, mmol/L, median (IQR)	3.78 (2.94, 4.57)	4.38 (3.63, 5.06)	−2.885	0.004
HDL‐C, mmol/L, median (IQR)	0.72 (0.52, 1.00)	1.02 (0.76, 1.37)	−3.476	0.001
LDL‐C, mmol/L, median (IQR)	2.15 (1.57, 2.84)	2.50 (1.97, 3.01)	−1.879	0.060

Abbreviations: BMI, body mass index; CKD‐EPI Sc, a equation of chronic kidney disease epidemiology collaboration base serum creatinine; HDL‐C, high‐density lipoprotein cholesterol; LDL‐C, low‐density lipoprotein cholesterol; Scr, serum creatinine; TC, total cholesterol; TG, triglyceride.

**Table 3:** Changes in biochemical indexes between baseline and Week 48.

Indexes	Baseline	Week 48	*t/z*	*p* value
BMI, kg/m^2^, mean ± SD	19.60 ± 2.85	19.80 ± 2.85	−0.310	0.757
CD4, cells/μL, median (IQR)	64.50 (35.75, 144.00)	242.00 (162.50, 356.00)	−5.811	< 0.001
CD4/CD8, median (IQR)	0.20 (0.12, 0.29)	0.38 (0.22, 0.50)	−4.350	< 0.001
Scr, μmoL/L, median (IQR)	57.25 (42.00, 65.25)	74.00 (62.00, 91.00)	−4.161	< 0.001
CKD‐EPI Scr, mL/min, median (IQR)	106.55 (71.55, 152.70)	69.68 (53.45, 100.91)	−3.212	0.001
Uric Acid, μmoL/L, median (IQR)	282.50 (204.25, 361.00)	369.00 (295.50, 458.50)	−3.039	0.003
TG, mmol/L, median (IQR)	1.78 (1.18, 2.12)	1.54 (1.27, 2.43)	−0.313	0.754
TC, mmol/L, median (IQR)	3.78 (2.94, 4.57)	4.26 (3.67, 4.74)	−2.438	0.015
HDL‐C, mmol/L, median (IQR)	0.72 (0.52, 1.00)	1.09 (0.88, 1.34)	−4.684	< 0.001
LDL‐C, mmol/L, median (IQR)	2.15 (1.57, 2.84)	2.21 (2.02, 3.03)	−1.888	0.059

Abbreviations: BMI, body mass index; CKD‐EPI Sc, a equation of chronic kidney disease epidemiology collaboration base serum creatinine; HDL‐C, high‐density lipoprotein cholesterol; LDL‐C, low‐density lipoprotein cholesterol; Scr, serum creatinine; TC, total cholesterol; TG, triglyceride.

### 3.4 Safety

Serum creatinine levels increased at 24 and 48 weeks, and the eGFR based on creatinine decreased at 24 and 48 weeks compared with baseline (all, *p* < 0.05). Uric acid levels increased at 24 and 48 weeks (*p* < 0.001, *p* = 0.003). Total cholesterol (TC) levels increased at 24 and 48 weeks (*p* = 0.004, *p* = 0.015). HDL‐C levels were significantly higher at 24 and 48 weeks than that at baseline (*p* < 0.05). There were no significant changes in body mass index (BMI), TG and LDL‐C from baseline to week 24 (Table 2) and week 48 (Table 3) (*p* > 0.05).

### 3.5 TB Efficacy

**Figure 3:**
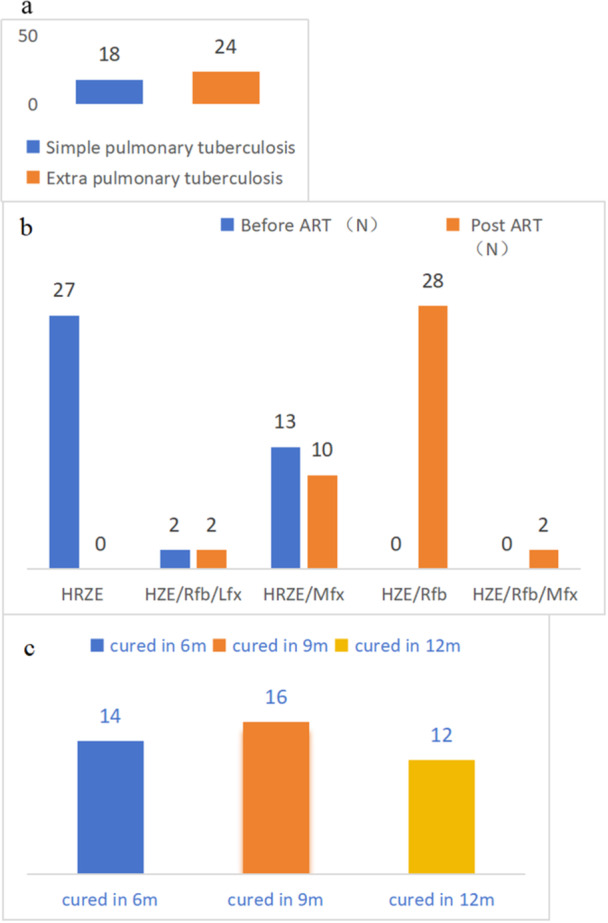
Efficacy of patients with tuberculosis.

The efficacy of anti‐TB treatment was 42.8% (18/42) in patients with pulmonary TB, and 71.1% (24/42) patients with extrapulmonary TB. The latter group included 9 patients with TB involving neck lymph nodes TB, 12 with abdominal lymph node involvement, and 3 patients with of intracranial TB. None of the patients exhibited TB drug resistance (Figure 3a).

Before ART initiation, 64.3% (27/42) of the patients were treated with HREZ, 4.8% (2/42) with HZE/Rfb/Lfx, and 40.0% (13/42) with HRZE/Mfx. After ART initiation, 66.8% (28/42) of the patients changed their anti‐TB regimen to HZE/Rfb. The anti‐TB regimen was adjusted to HZE/Rfb/Mfx in 4.8% (2/42) and HZE/Rfb/Lfx in 4.8% (2/42) of patients, resulting in a total of 32 patients receiving rifabutin‐based anti‐TB regimens after ART (Figure 3b). The reason for adjustment of the TB regimen was because lamivudine and dolutegravir are covered by medical insurance in our province, and dolutegravir only needs to be administered at a dosage of 50 mg OD, thereby reducing the economic burden. The anti‐TB regimen was based on a rifampicin anti‐TB regimen, and dolutegravir was administered at a dosage of 50 mg BD.

Most patients with TB tested positive for pathogens (90.5%, 38/42). The etiology in most patients was negative after 6 months of anti‐TB treatment, and all patients were negative after 12 months of anti‐TB treatment (Table 4).

**Table 4:** Etiological examination.

Etiological examination(N)	Baseline	2 m	5 m	6 m	12 m
Positive	38	7	0	0	0
Negative	4	35	42	37	42
Not detected	0	0	0	5	0
IRIS	14				

After 6 months of anti‐TB treatment, 33.3% (14/42) were cure; 31.0% (16/42) were cured after 9 months; and the remaining 28.6% (12/42) were cured after 12 months (Figure 3c). A total of 26.2% (11/42) of patients had immune reconstitution inflammatory syndrome (Table 1).

### 3.6 Adverse Events

There were 2 cases of transient dizziness; 4 cases of headache; no cases of allergy, diarrhea, vomiting, diarrhea or other adverse events; no cases of serious adverse events: death. Regarding safety monitoring of anti‐tuberculosis drugs, one patient had slightly elevated aspartate aminotransferase and alanine aminotransferase at 24 weeks, wherein liver function returned to normal after liver protection therapy, while no patients experienced severe liver function damage.

## 4 Discussion

Although TB/HIV requires anti‐TB treatment at the same time as ART, the interactions of ART and TB drugs complicates the treatment. There are few data on the application of DTG/3TC or CDTG + 3TC combination therapy with rifamycins as the first‐line TB drugs. More clinical studies are needed to elucidate the efficacy and safety of phase II in patients with HIV and TB.

A real‐world study in Guangxi [9] examined the efficacy of DTG + 3TC qd administered with food in TB/HIV co‐infected patients on stable rifampicin treatment All participants achieved virologic suppression at week 24 (83.3%). The overall viral suppression rate in our study was 73.8% at week 24, which was lower than that of the real‐world data in southern China [10] and similar to that of a study in Guangxi. The possible reasons for this are that HIV co‐infection with TB affects the duration of ART, and the occurrence of immune reconstitution inflammatory syndrome in patients may affect the control of HIV in the short term. However, the overall viral suppression rate at week 48 was 95.2%, which was close to the real data in southern China and studies in Germany and Spain [9, 11]. There were two cases of virological treatment failure in this study. One patient could not take HIV drugs regularly due to TB encephalitis paralysis and did not have family care; another patient was an older adult and could not take care of himself and missed taking HIV drugs 2‐3 times per month.

The viral suppression rates of the CD4 + T‐cell ≥ 200/mL and CD4 + T‐cell < 200/mL groups were 100.0% and 69.4% at 24 weeks, and 100.0% and 94.4% at 48 weeks, respectively. Consistent with a real‐world study in Southwest China, DTG/3TC was used for the initial treatment study [10]. In a real‐world study in Guangxi [9] of treatment‐naive patients with high disease burden (baseline HIV RNA > 500,000 cp/mL) receiving DTG + 3TC, the virologic suppression rates at 24 and 48 weeks were 46.7% and 80.0%, respectively. The incidence of HIV RNA ≥ 500,000 cp/mL was 28.5% and 100.0% at 24 and 48 weeks, respectively. The possible reasons for the difference between the two studies are that the data were from single‐center with small sample sizes. The efficacy of DTG + 3TC in patients with high HIV load needs to be further studied in multicenter and large‐sample studies.

Data of two multi‐center cohorts of patients with HIV infection in Spain [14] showed that patients treated with DTG + 3TC had increases in CD4 + T‐cell counts at 24 and 48 weeks compared with baseline, with a slight increase in the CD4 + /CD8+ ratio. The CD4+ cell counts at weeks 24 and 48 were significantly higher than those at baseline (*p* < 0.05), and the CD4 + /CD8+ ratio also slightly increased. However, the difference was not statistically significant, which may be related to the small number of patients and short observation time.

Seven patients had abnormal baseline creatinine levels, and the dose of 3TC did not need to be adjusted according to the eGFR. Serum creatinine levels increased at 24 and 48 weeks, and the eGFR based on creatinine decreased at 24 and 48 weeks compared with baseline. The clinical observation and study of the renal safety of DTG + 3TC in the long‐term use of large samples in multi‐centers is still needed.

The body mass index in patients at 24 and 48 weeks in this study were slightly higher than those at baseline; however, the differences were not statistically significant, which was consistent with the above studies [12]. Although there was no statistical significance in the increase in body mass index, the possible reason is that our study had a small sample size. Conducting a multi‐center and large sample observation study for a longer period of time is necessary to understand whether the increase in body mass index could have adverse effects on the health of patients, such as cardiovascular and cerebrovascular diseases.

Drug safety and ease of administration affect patient compliance. Consistent with other findings, at 48 weeks of treatment, patients had elevated creatinine levels and decreased eGFR based on creatinine, uric acid without renal function impairment [13, 14]; this may be related to DTG inhibiting mailed cation transport protein 2 outside the proximal tubular cell base of the kidney, resulting in a mild, non‐progressive serum creatinine elevation [15, 16]. Uric acid elevation is due to the effect of pyrazinamide on uric acid elevation in anti‐tuberculosis regimen. However, the increase range was within the normal range and had nothing to do with DTG.

The results of the GEMINI 96‐week study showed changes in the high‐density lipoprotein [17], with increased levels at weeks 24 and 48 compared with baseline (both *p* < 0.05), which was consistent with the results of the current study. The mechanism of DTG + 3TC in the treatment of AIDS patients with elevated HDL is still unclear and needs further clinical research. Cardiovascular disease is the most common non‐HIV‐related disease in patients with HIV; for which total cholesterol and elevated LDL‐C levels are independent risk factors [10, 18]. In Chinese patients with HIV, the dyslipidemia incidence is 75.6%, primarily manifested by triglyceride and LDL‐C increases. In this study, dyslipidemia at 48 weeks was mainly manifested as elevated low density lipoprotein, high density lipoprotein, and total cholesterol and significant changes in total cholesterol, which was consistent with findings in the existing literature. In the process of using DTG antiretroviral therapy, blood lipid levels should be closely monitored and corresponding intervention measures should be taken.

In a study from Botswana [19]of patients with TB/HIV treated with DTG‐based ART regimens, high rates of VL suppression (> 92%) were observed across all ART regimens. Of patients receiving the DTG regimen, 90.9% had good TB treatment outcomes, compared with 88.3% of those receiving the non‐DTG regimen. The study showed that 33.3% (14/42) of the patients were cured at 6 months, 31.0% (16/42) were cured at 9 months, and 28.6% (12/42) were cured at 12 months after anti‐TB treatment. Consistent with the study, it indicates that DTG + 3TC could effectively inhibit HIV virus in patients with AIDS with tuberculosis.

There were no serious adverse events in this study, consistent with reports in the literature [9].

This study has some limitations. This study was a single‐center, small‐sample study, lacking control group and study data on whether later TB infection was reactivated. The study of DTG + 3TC in HIV/TB patients complicated with tuberculosis requires multi‐center and larger sample study, so as to make the study more complete and perfect.

## 5 Conclusion

DTG + 3TC could effectively inhibit HIV replication and improve immune function in patients with HIV/TB and showed good safety, which might be one of the best treatment options in patients with HIV infection/AIDS complicated with TB.

## Author Contributions

**Jinhong He:** conceptualization, resources, writing – original draft. **Xiangxi He:** data curation, resources, software. **Xiaoxin Xie:** data curation, supervision. **Yanhua Fu:** validation. **Xingxing Luo:** methodology. **Yinshuang Peng:** methodology. **Bin Xu:** data curation. **Hai Long:** conceptualization, writing – review and editing.

## Ethics Statement

The study was performed according to the principles of the Declaration of Helsinki, and prior approval was obtained from the Institutional Review Board of Research Center (202206: approval date: 2022.06). Informed consent was obtained from all participants prior to their enrollment in the study.

## Conflicts of Interest

The authors have no relevant affiliations or financial involvement with any organization or entity with a financial interest or financial conflict with the subject matter or materials discussed in this manuscript.

## Data Availability

The authors confirm that the data supporting the findings of this study are available in the article.
